# Prospective study of the neurotopographic adequacy of transverse incision in Lichtenstein inguinal hernioplasty

**DOI:** 10.1097/MD.0000000000005335

**Published:** 2016-11-04

**Authors:** Carlos J.L. Mendes, Rodrigo A. Silva, Daniel P.A. Neto, Isabela Brianti, Kassem Saleh, Mirna D. Barros, Sergio Roll, Adhemar M. Pacheco

**Affiliations:** a Surgery Department; b Morphology Department; c School of Medicine, Santa Casa de Sao Paulo School of Medical Sciences, São Paulo/SP, Brazil.

**Keywords:** abdominal, abdominal wall, anatomy, hernia

## Abstract

Lichtenstein technique requires identification of the iliohypogastric, ilioinguinal, and genital branch of the genitofemoral nerves.

The aim of the study was to verify if the transverse incision is suitable for identification of the iliohypogastric, ilioinguinal, and genital branch of the genitofemoral nerves.

This study included 29 patients who underwent hernioplasty, and also 10 dissections of the inguinal regions from 5 cadavers. The anthropometric measurements included: incision size (IS) and topography, pubic angle (PA), body mass index (BMI), and the distance from the pubis to the incision and bi-iliac crest plane. The correlations between variables of interest and the ability to identify the nerves were assessed.

Measures of height (*P* = 0.108), BMI (*P* = 0.343), and abdominal circumference (AbC) (*P* = 1.000); the correlations between incision IS and PA (*r* = −0.17, *P* = 0.406), IS and BMI (*r* = 0.56, *P* = 0.002), IS and AbC (*r* = 0.56, *P* = 0.002); incision and pubic heights (*r* = −0.26, *P* = 0.174); patient height and PA (*r* = −0.33, *P* = 0.092). The associations between these measures were: BMI (*P* = 0.136), AbC (*P* = 0.104), PA (*P* = 0.641), and IS (*P* = 0.399). The rates of successful nerve identification in patients and corpse were: iliohypogastric—29 (29)/9 (10), 100% (*P* = 0.147); ilioinguinal—29 (29)/10 (10), 100%; and genital branch of the genitofemoral nerve—26 (29)/9 (10), 89.7/80% (*P* = 0.488).

The transverse incision permitted identification of the nerves for Lichtenstein hernioplasty.

## 1 Introduction

Although inguinal hernioplasties are one of the most frequently performed surgical procedures, complications may occur. Relapse and pain in the late postoperative period are some of the most frequent complications. Several modifications to inguinal hernia repair techniques have been introduced, including the use of biocompatible implants, with the aim of reducing recurrence rates.

The use of prostheses, particularly the polypropylene mesh proposed by Giulio Natta in 1954, has been progressively disseminated and have been employed in the form of a sheet on the inguinal floor.^[1,2]^ In the past 20 years, the frequent use of prostheses in surgical treatment of inguinal femoral hernias has resulted in decreased recurrence rates; however, a larger incidence of postoperative pain has been observed, which has important repercussions on patient quality of life.^[3]^

In 1998, Heise and Starling^[4]^ analyzed the postoperative period of 20 patients who required partial or total prosthesis removal to treat chronic pain after hernioplasty, describing such pain as “mesh inguinodynia.”

Magee^[5]^ first described “genitofemoral causalgia” in 1942, demonstrating that the factors related to pain could be avoided with the identification, dissection, and preservation of the nerves of the inguinal region.

Inguinal hernioplasty, proposed by Lichtenstein et al^[6]^ in 1989 and referred to as tension-free hernioplasty with use of mesh, emphasizes the necessity of identifying the iliohypogastric, ilioinguinal, and genital branch of the genitofemoral nerve, independent of the incision type.^[6,7]^

Whereas there are numerous techniques for correction of inguinal hernias, tension-free hernioplasty with prosthesis under local anesthesia predominates, based on a systematic review from the Cochrane Library in 2001 and the European Hernia Society guidelines on the treatment of inguinal hernia in adult patients.^[8,9]^

According to the International Association for the Study of Pain, the lesion, dissection, nerve compression, and contusion, and also the use of electrocautery, prostheses, metal clasps, and sutures for mesh fixation contribute to unpleasant outcomes.^[10]^

In principle, several factors may contribute to the difficulty in identifying nerves in this region; however, anthropometric and biometric data are scarce regarding the neurotopographical adequacy of the transverse incision recommended by the Lichtenstein technique, for identification of the iliohypogastric and ilioinguinal nerves, and genital branch of the genitofemoral nerve.

Thus, the objective of this study was to assess if the transverse incision in the inguinal region during the Lichtenstein hernioplasty is suitable for identification of the iliohypogastric and ilioinguinal nerves, and genital branch of the genitofemoral nerve.

## 2 Methods

A total of 29 patients with inguinal hernias underwent surgery by the Lichtenstein technique.^[7]^ Bilateral dissections of the inguinal region were also performed in 5 adult cadavers from the Morphology Department of the Santa Casa de São Paulo School of Medical Sciences.

The study was approved by the Research Ethics Committee (opinion number 1.300.651).

### 2.1 Inclusion criteria

The inclusion criteria were as follows:

1. Adult men between 18 and 85 years of age.
2. Body mass index (BMI) less than 35 kg/m^2^.
3. Patients with unilateral inguinal hernia, types II or III, according to Nyhus classification.
4. Physical condition based on American Society of Anesthesiology (ASA) criteria of I, II, or III.

### 2.2 Exclusion criteria

The exclusion criteria were as follows:

1. Patients with recurrent bilateral inguinal hernia, either incarcerated or strangulated.
2. Chronic users of analgesics, corticosteroids, antidepressants, anxiolytics, anticonvulsants, illicit drugs, and alcohol.
3. Patients with fibromyalgia.
4. Patients with previous surgery of the inguinofemoral region.
5. Patients with benign prostatic hyperplasia, not released after urological evaluation or treated by prostatic surgery.
6. Patients categorized as ASA IV and V.

### 2.3 Cadaver inclusion criteria

The Cadaver inclusion criteria were as follows:

1. Adult men between 18 and 85 years of age.
2. Body mass index lower than 35 kg/m^2^.

### 2.4 Cadaver exclusion criterion

The Cadaver exclusion criterion was as follows:

1. Evidence of previous surgery in the inguinal region.

### 2.5 Surgical technique

The patients received a subarachnoid block. After preparing the surgical field, the location of the incision was determined and a straight line marked from the transversal to the inguinal regions with a sterile dermographic pen before the incision was made. After opening the aponeurosis of the external oblique muscle, the iliohypogastric and ilioinguinal nerves and spermatic cord were identified. The cremaster fibers of the spermatic cord were exposed to dissect the hernial sac, in indirect hernias, and to identify the genital branch of the genitofemoral nerve in the posterolateral topography of the cord. The Lichtenstein hernioplasty technique was used, as described previously.^[7]^

At the end of the surgery, the following measures were taken:

1. Weight (kg), height (m), and BMI (kg/m^2^).
2. Abdominal circumference (AbC) (cm) at the height of the umbilicus.
3. Herniation laterality (right, left, or bilateral).
4. The surgical team was questioned on their ability to identify the three nerves.
5. Measurement (cm) of the triangle bordered by the edges of the pubic symphysis to the right and left anterior superior iliac spines, in the anterior superior iliac spine plane.

The distance measurements were as follows:

1. AB: From the pubic symphysis to the anterior superior iliac spine, to the right or left.
2. BC: From the anterior superior iliac spine to the mid line—plane that passed by the right and left anterior superior iliac spines.
3. AC: From the pubic symphysis to the plane that passed through the anterior superior iliac spines.
4. ED: Incision size to the right or left.
5. AD: Incision height—distance from the pubic symphysis to the incision.
6. ÂBB: Pelvic opening angle (degrees).

### 2.6 Dissection of human cadavers

**Figure 1:**
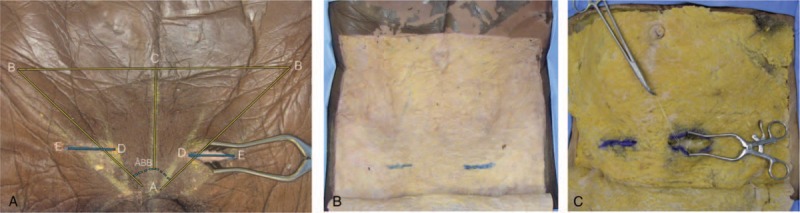
Anterior view: anthropometric measurements, incisions, and projections. A, Skin incision; autostatic retractor: left side. AB = triangle edge, BB = triangle edge, AC = distance from the pubic symphysis to the plane of the anterior superior iliac spines, ED = incision size, right or left, AD = incision height—distance from the pubic symphysis to the incision, ÂBB = pelvic opening angle (in degrees). B, Incision “window.” In blue, incision in the subcutaneous, projection of the incision of the skin. C, In blue, subcutaneous incision. Autostatic retractor, exposing the superficial epigastric vein, tractioned with wire by Kelly forceps.

The same measurements were taken after bilateral dissections of the inguinal regions of the 5 cadavers. A surgeon was invited to demarcate the incision of transverse inguinotomy, with the objective of identifying the nerves in question. A skin incision was made to the subcutaneous layer, followed by demarcation of the same with a dermographic pen and dissection of the skin to expose the subcutaneous layer “in window.” Next, the aponeurosis of the external oblique muscle was demarcated in the projection of the dimensions of the skin incision, followed by identification of the superficial epigastric vein of the external oblique muscle, superficial inguinal ring, and spermatic cord (Fig. 1).

**Figure 2:**
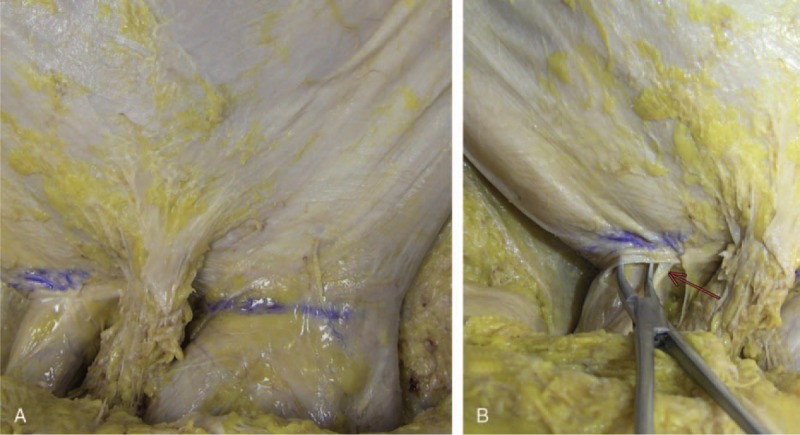
Anterior view: projection of the incision in the aponeurosis. A, Demarcation in blue of the projection of the incision in the aponeurosis in both inguinal regions. B, Right inguinal region. Forceps exposing the superficial inguinal ring. Arrow, in red, pointing to the ilioinguinal nerve. In blue, projection of the incision in the aponeurosis.

Dissection by detachment of the subcutaneous tissue to expose the superficial external oblique muscle aponeurosis, superficial inguinal ring, and spermatic cord was performed using the same skin window incisions (Fig. 2).

### 2.7 Statistical analysis

The variables were analyzed descriptively as means, SDs, medians, minimum and maximum values for quantitative variables, and absolute (n) and relative (%) frequencies for categorical variables.

Mann–Whitney tests were used for comparisons between patients and cadavers. The associations between the study group and categorical variables were evaluated by Fisher exact test.

The correlation between the measures of interest was assessed using Pearson correlation coefficient.

A multiple logistic regression model was used to evaluate the association between BMI, AbC, pubic angle (PA), and incision size (IS) with the ability to identify the three nerves of interest.

The significance level was 0.05 (α = 5%) in all the statistical tests and PASW Statistics for Windows, version 18.0 (SPSS, Inc., Chicago, IL), was used for analysis.

## 3 Results

Of 30 patients initially selected, 1 was excluded for not presenting clinical conditions at the time of surgery. The ages of the 29 included patients ranged between 18 and 82 years, with a mean and median of 45.5 ± 18.3 and 49 years, respectively. Regarding comorbidities, 17.2% of the patients were smokers and 13.8% were obese. According to Nyhus classification, 55.2% of patients were type II, 6.9% were IIIA, 34.5% were IIIB, and 3.4% were IIIC. The surgical time ranged between 30 and 130 minutes, with a mean and median of 70.8 ± 21.7 and 70 minutes, respectively.

Among the patients, 58.6% were operated on the right side, whereas all cadavers were dissected bilaterally. There was a statistically significant difference between groups regarding the distribution of weight (*P* = 0.043). In this study, the patients had a lower median weight than the cadavers.

No significant differences were found between groups regarding the distributions of height, BMI, and AbC.

Descriptive analyses of the participant measurements according to study group are shown in Table 1. A significant difference was found between groups regarding the distribution of IS (*P* = 0.022). The patients presented a larger median IS than that of the corpses. A significant difference was also observed between the groups regarding the distribution of the AC measurements (*P* = 0.002). The patients had a lower median AC measurement than that of the corpses. No significant differences were found between the groups regarding the distributions of other measurements.

**Table 1:**
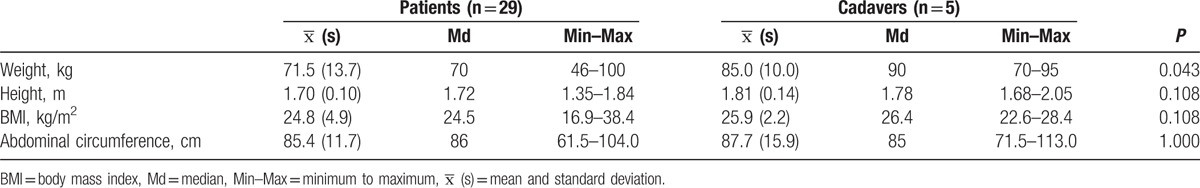
Anthropometric measures according to participant study group.

Descriptive analyses of the surgical measurements of the participants according to the study group are shown in Table 2. No association was found between the study group and the isolated identification of the iliohypogastric, ilioinguinal, and genitofemoral nerves, and the correct identification of all 3 nerves.

**Table 2:**
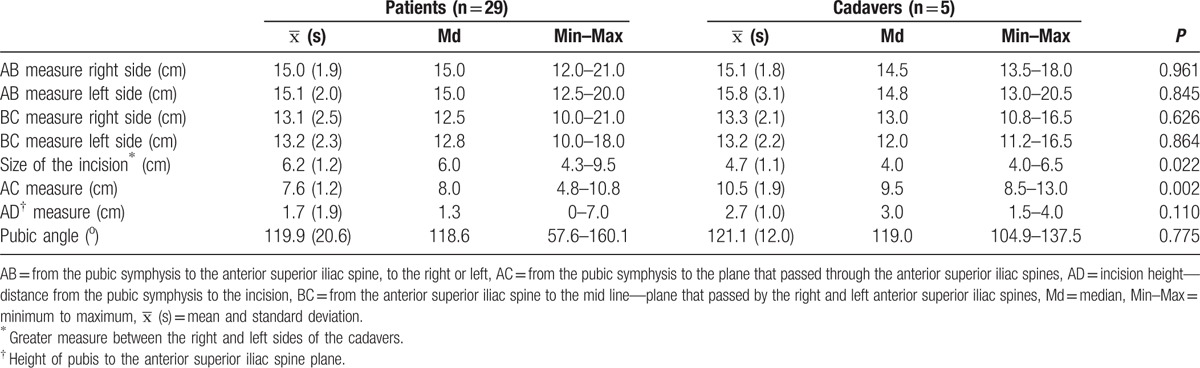
Surgical measures according to participant study group.

Descriptive analyses of the nerves investigated according to study group are shown in Table 3.

**Table 3:**
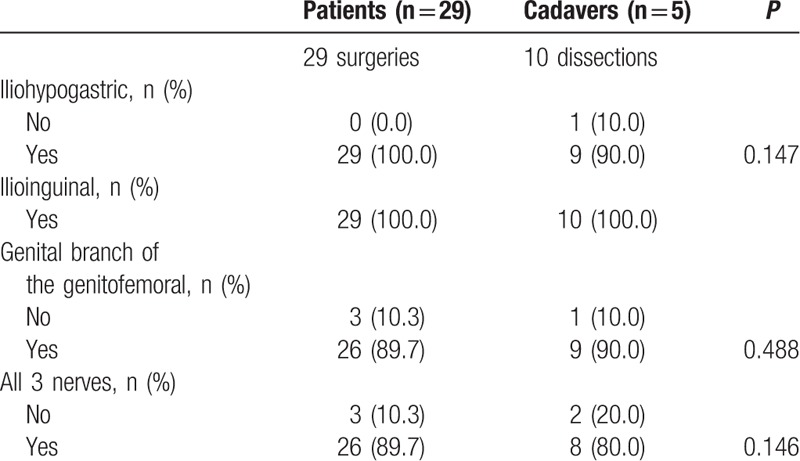
Identification of nerves according to study group.

Correlations between the variables of interest in the group of patients were evaluated (n = 29), and the interpretation of the magnitude of the correlation was determined using the classification proposed by Evans.^[11]^ According to this empirical classification, *r* < 0.20 was considered a very weak correlation; between 0.20 and 0.39, weak; between 0.40 and 0.59, moderate; between 0.60 and 0.79, strong; ≥0.80, very strong.

A very weak and nonsignificant inverse correlation was found between IS and PA (*r* = −0.17, *P* = 0.406). Direct, moderate, and significant correlations were observed between IS and BMI (*r* = 0.56, *P* = 0.002) and AbC (*r* = 0.56, *P* = 0.002).

An inverse, weak, and nonsignificant correlation was found between incision height and the height of the pubis to the anterior superior iliac spine plane (*r* = −0.26, *P* = 0.174).

An inverse, weak, and nonsignificant correlation was found between patient height and PA (*r* = −0.33, *P* = 0.092) (Table 4).

**Table 4:**
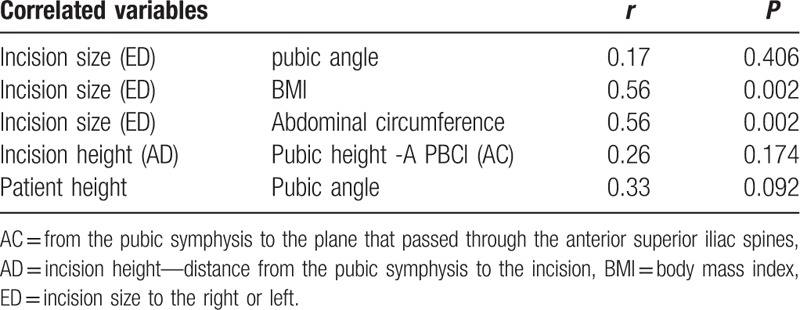
Correlation between variables.

A multiple logistic regression model was adjusted to evaluate the association of BMI, waist circumference, PA, and IS, with the identification of the 3 nerves investigated in the patients and cadavers. There was no evidence of a significant association between the identification of the nerves with the explanatory variables evaluated (*P* > 0.05 in all variables) (Table 5).

**Table 5:**
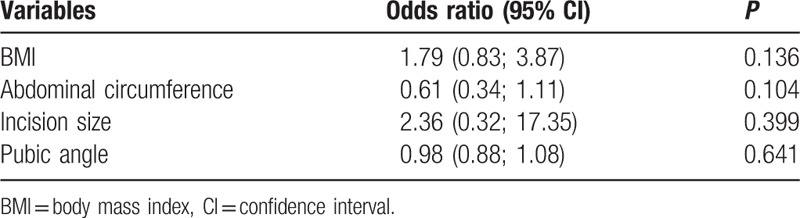
Multiple logistic regression model evaluating the association between identification of the three nerves and the variables of interest.

## 4 Discussion

In 2003, Callesen^[12]^ reported that severe pain that occurs from 1 to 4 weeks in the postoperative period was a predictive factor for pain up to 1 year.

In a 2006 prospective multicenter study, Alfieri et al^[13]^ evaluated eleven institutions, including 955 patients and 1050 hernioplasties with prostheses, surveying the surgeons on the identification and preservation of the iliohypogastric, ilioinguinal, and genitofemoral nerves. They concluded that the preservation of nerves was an important factor in reducing the incidence of postoperative pain; however, they did not evaluate the type of incision.^[13]^

In a 2011 prospective cohort study involving 781 hernioplasties reported by Reinpold et al,^[14]^ 63.1% and 36.9% of operations were performed by the Lichtenstein technique and Shouldice technique, respectively. The incidences of chronic pain after 6 months to 5 years were 16.5% and 16.1%, respectively, and the combination of neurolysis of the ilioinguinal nerve and use of prosthesis was associated with chronic pain.

In 1997, Luijendjik et al^[15]^ assessed nerve entrapment of the inguinal region, and also the formation of neuromas (2.1%), paresthesia (25.1%), and the length of the Pfannenstiel transverse incision as a risk factor (*P* = 0.02), and concluded that identification of the ilioinguinal nerve is mandatory.

**Figure 3:**
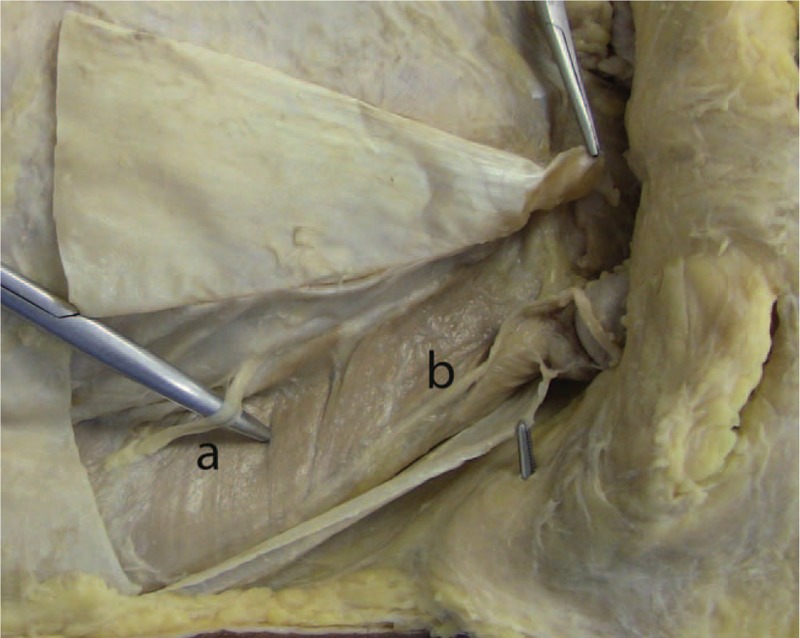
Anterior view: right inguinal region. A, Iliohypogastric nerve penetrated the myoaponeurotic sheath of the rectus abdominis muscle more cranially. B, Ilioinguinal nerve.

Countless studies have evaluated the topography of the iliohypogastric and ilioinguinal nerves, and the genital branch of the genitofemoral nerve, especially when one considers their origins and inter-relationship in the sacrococcygeal plexus, in both adults and children.^[16]^ The topographical distribution of the iliohypogastric nerve on the abdominal wall presents a high index of anatomical variation. It becomes visible on the lateral edge of the psoas major muscle, 5.1 to 9.2 cm cranially to the posterior superior iliac spine, pierces the aponeurosis of the transverse muscle of the abdomen above the iliac crest an average of 3 cm in the medial direction of the anterior superior iliac spine, penetrates the layers of the transverse abdominis and internal oblique muscles, migrating toward and in the caudal direction, an average of 4 cm cranial and parallel to the inguinal ligament, covered by the aponeurosis of the external oblique muscle. During the dissection of cadaver number 4, the path of this nerve was exactly as described; however, it was outside the area of dissection offered by the transverse incision due to the fact that the nerve penetrated the myoaponeurotic sheath of the rectus abdominis muscle more cranially, as shown in Fig. 3.^[17]^

The ilioinguinal nerve, in turn, becomes visible at the edge of the psoas muscle, 4.4 to 8.6 cm cranially to the posterior superior iliac spine and caudally to the iliohypogastric nerve. The nerve passes through the transversus abdominis 1 cm above the iliac crest and 2 cm anteriorly to the iliohypogastric nerve. The nerve penetrates the oblique internal muscle 4 to 5 cm medially to the anterior superior iliac spine.^[12,13]^ After a short course through the inguinal canal, the nerve comes medially to the spermatic cord in the superficial inguinal ring (72%) or sideways (10%) to the spermatic cord or round ligament. It follows the spermatic cord for 2 to 4 cm. Our study showed that the transverse incision was able, topographically, to identify the ilioinguinal nerve in the study group.^[17]^

The genital branch of the genitofemoral nerve presents in the retroperitoneal space, tapping into the abdominal wall near the deep inguinal ring. The femoral branch passes below the inguinal ligament and follows the external inguinal ring. Our study demonstrated that the genital branch of the genitofemoral nerve was not found in 10% of the study group, although the nerve trajectory was in the area of the transverse incision approach. We evaluated the presence of anatomical variations, or inadvertent dissection followed by unnoticed lesions.^[17]^ The relevance of this subject was described in a meta-analysis by Barazanchi et al^[18]^ in 2016, who concluded that efficient routine neurectomy of the ilioinguinal nerve is safe; however, neurectomy of the genitofemoral nerve was not included in the study.^[18]^

Regardless of the cosmetic advantages of the transverse incision, the findings of the current anatomical study demonstrated that it was possible to identify the 3 nerves despite the large variation in IS. The difference in IS between groups was due to the technical difficulty in handling herniations without damaging the nerves. The operative difficulty due to the characteristics of the herniation, inadvertent dissection or anatomical variations, can be associated with the nonidentification of nerves, such as the genital branch of the genitofemoral nerve.

Although there were differences of 102° and 91.4° among patients and cadavers, respectively, the PAs did not interfere with the ability to identify the nerves. Although there was an increase in angle, there was no increase in the IS.

Body mass index and the AbC were associated with slight increases in IS. The distance from the incision to the iliac crest plane, which in theory could create a more cranial position, showed no correlation.

The results of anatomical-clinical studies such as the current survey justify opinions; however, the participants included in this study did not include the extremes of obesity or greater ranges in height. Thus, further study is necessary to confirm the observations of this study.

## 5 Conclusions

Based on the anthropometric measurements in this study population, the transverse incision is suitable for topographical identification of the iliohypogastric, ilioinguinal, and genital branch of the genitofemoral nerves, a finding that supports the surgeon's decision to opt for this access. According to our study, the transverse incision is an appropriate choice for both cosmetic and neurotopographical reasons, to minimize postoperative complications, particularly inguinodynia, due to incorrect mesh placement on these nerves.
